# A breath of fresh air in microbiome science: shallow shotgun metagenomics for a reliable disentangling of microbial ecosystems

**DOI:** 10.20517/mrr.2021.07

**Published:** 2022-02-25

**Authors:** Gabriele Andrea Lugli, Marco Ventura

**Affiliations:** ^1^Laboratory of Probiogenomics, Department of Chemistry, Life Sciences and Environmental Sustainability, University of Parma, Parma 43124, Italy.; ^2^Microbiome Research Hub, University of Parma, Parma 43124, Italy.

**Keywords:** Metagenomics, microbiota, bioinformatics, microbial DNA sequencing

## Abstract

Next-generation sequencing technologies allow accomplishing massive DNA sequencing, uncovering the microbial composition of many different ecological niches. However, the various strategies developed to profile microbiomes make it challenging to retrieve a reliable classification that is able to compare metagenomic data of different studies. Many limitations have been overcome thanks to shotgun sequencing, allowing a reliable taxonomic classification of microbial communities at the species level. Since numerous bioinformatic tools and databases have been implemented, the sequencing methodology is only the first of many choices to make for classifying metagenomic data. Here, we discuss the importance of choosing a reliable methodology to achieve consistent information in uncovering microbiomes.

During the past two decades, the evolution of DNA sequencing technologies has allowed the gathering of a vast amount of genetic material, laying the foundation to study complex microbial communities, also called microbiomes. At the dawn of the metagenomic classification era, it was necessary to distinguish each taxon based on their 16S rRNA gene sequence to unveil the composition of the bacterial communities inhabiting specific environments^[1]^. However, for many years, a significant portion of the microbiome has been ignored using this approach, such as archaea, fungi, protists, and viruses [Figure 1]. Nonetheless, to date, 16S rRNA microbial profiling is still a widely used methodology to dissect the composition of bacterial communities. To make up for its weakness, it is usually compensated by additional sequencing steps, e.g., internal transcribed spacer sequencing for fungal community identification^[2]^. Another weakness of this methodology is the depth of results that rely on the *in silico* generation of operational taxonomic unit (OTU) or amplicon sequence variant (ASV). While OTUs are usually used to classify the sequencing outputs at the bacterial family or genera level, ASVs claim to reach the classification at the species level. Unfortunately, using short-read sequencing targeting one or two variable regions of the 16S rRNA gene is not enough to reach the classification at the species level for all microorganisms. For example, variable regions between microorganisms can reach very high similarities values in both pathogenic bacteria such as *Escherichia coli* and *Shigella* spp.^[3]^ and commensal bacteria such as species of the genus *Bifidobacterium*^[4]^. Thus, caution is necessary for the interpretation of 16S rRNA profiles when blindly using bioinformatic tools. Nowadays, longer length sequencing reads have been achieved, improving the accuracy of species detection by accomplishing the complete length of the 16S rRNA gene. For example, using Oxford Nanopore Technologies, the reconstructed ASVs will improve the resulting microbial profiling with respect to the same analysis performed using short-read sequencing systems such as Illumina technology. In the same fashion, PacBio single-molecule real-time (SMRT) technology is also capable of full-length 16S rRNA gene sequencing, and it has been proposed as an alternative approach to target all nine variable regions of the 16S rRNA gene^[5]^.

**Figure 1:**
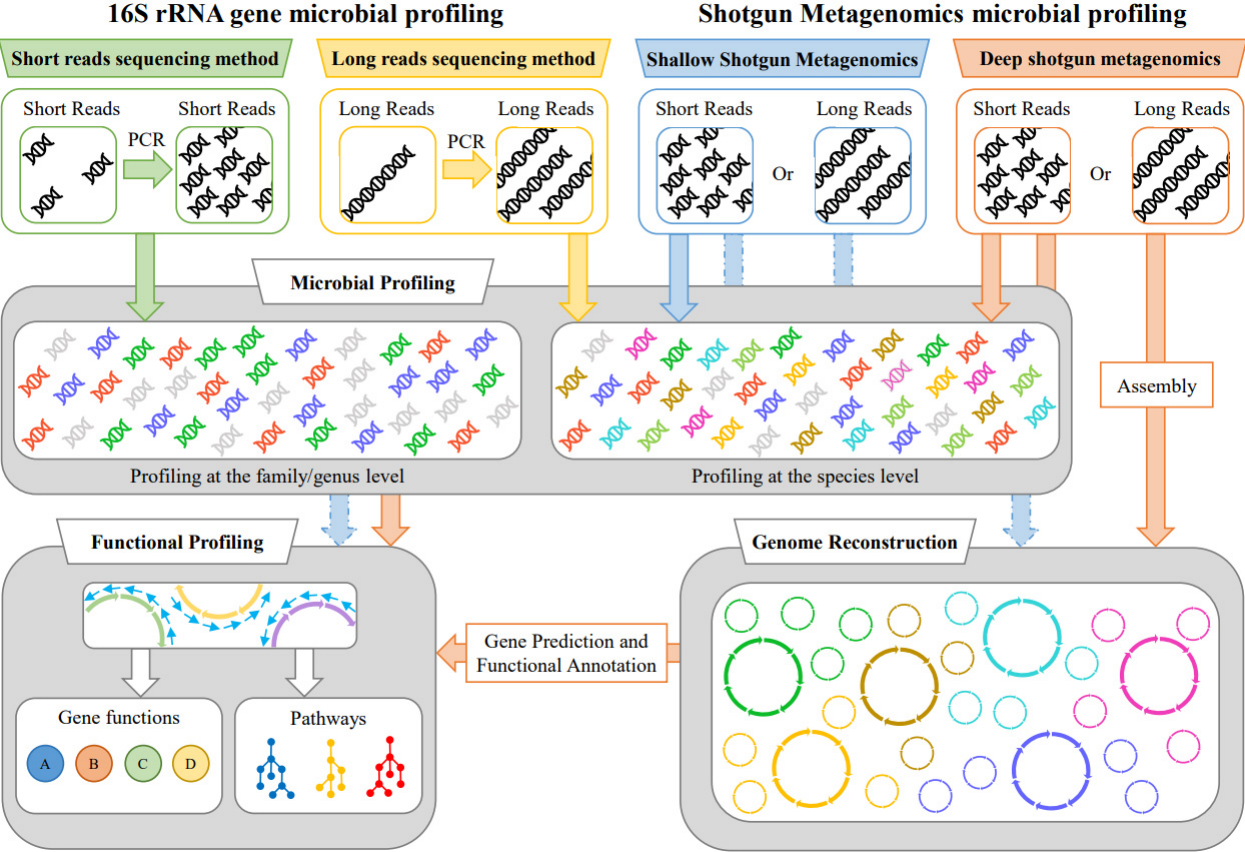
Schematic representation of the methodologies based on 16S rRNA gene microbial profiling and shotgun metagenomics.

However, long-read sequencing technology cannot counteract other issues related to 16S rRNA gene profiling, such as the different number of rRNA loci distributed among genomes of the same genus and numerous taxa of the same species. Data normalization procedures are usually applied to balance the identified amount of rRNA, resulting in approximations of the actual abundance of each microbial taxon that may result in over- or under-estimation of the real microorganism abundance. Besides, the amplification protocol of metagenomic marker-based profiling may favor the amplification of contaminants, a notion that should not be underestimated in the interpretation of the results^[6]^. Moreover, the PCR amplification protocol represents a significant source of bias, generating PCR artifacts such as chimeras and heteroduplex molecules^[7]^. Furthermore, long-read technologies such as Oxford Nanopore and SMRT technology display a higher error rate compared to short-read sequencing systems, representing a serious issue in a reliable taxonomy assignment of microorganisms. It is now crucial to provide metagenomic datasets that can be compared in following up projects by the scientific community. Metagenomic projects will benefit from including microbial profiles previously analyzed by other groups to validate their results and compare microbiomes retrieved from other environments/conditions. Furthermore, the re-analysis of the DNA sequences from previous experiments that can be compared with new metagenomic datasets can also allow gathering a number of samples that could not be otherwise collected in a single study. Unfortunately, data obtained through different 16S rRNA gene profiling studies are not easy to compare due to the absence of a consensus standard in 16S rRNA microbial profiling protocols. In this context, so many different primers aiming at amplifying different variable regions are used, making it difficult to distinguish actual changes from profiled samples to problems related to the different specificity of distinct amplification methodologies^[8]^.

Based on the limitation of the short-read achieved in 16S rRNA gene profiling assays, alternative DNA sequencing strategies have been proposed to achieve more reliable information and avoid misclassification of microbes forcing re-analysis. Thus, the DNA sequencing of the whole microbial communities present in a biological sample, a procedure that is also called shotgun metagenomics, has been used to remove the amplification of marker genes, with the consequent reconstruction of a complete microbiome and the generation of data that are easy to compare between different datasets^[9]^. The main advantage of this approach is the ability to achieve the microbial composition of a microbiome in a single DNA sequencing step, including the makeup of bacteria, archaea, protists, and fungi [Table 1]. Furthermore, based on the sequencing depth, the taxonomic classification of the sequenced reads is only a fraction of the information that can be acquired. Chromosomal sequence reconstruction and functional annotation of microorganisms harbored in the biological samples are clear examples of how shotgun metagenomics can be more informative than metagenomic analysis based on the amplification of microbial marker genes. On the other hand, shotgun metagenomic sequencing is more expensive than 16S rRNA gene profiling. Thus, it is understandable that small research groups interested in screening microbial communities alone continue to choose 16S profiling due to their low budget, especially bearing in mind that it is crucial to have an adequate number of samples to achieve solid results based on statistical significance. Nonetheless, the computational power required to analyze shotgun metagenomics data is much heavier than that of 16S rRNA gene profiling, and advanced bioinformatic skills are necessary to manage the analysis steps. However, under specific circumstances, even shotgun metagenomics may not detect certain microorganisms from challenging samples, such as sub-dominant microorganisms or within samples dominated by a large amount of host DNA in host-related environments. In these circumstances, a DNA filtering step or a targeted DNA approach is mandatory^[10]^; otherwise, an even deeper shotgun sequencing is necessary, increasing the costs of these analyses. In this context, the hybridization capture targeting of the 16S rRNA gene, or other molecular markers, could be a complementary strategy to explore the microbial community at the species level^[11]^.

**Table 1:** Metagenomic strategies in uncovering the microbiota taxonomy

	**16S rRNA short-read sequencing**	**16S rRNA long-read sequencing**	**Shallow shotgun metagenomic sequencing**	**Shotgun metagenomic sequencing**
**DNA pre-amplification protocol**	Yes	Yes	No	No
**Sequencing depth (number of reads)**	~30,000	~30,000	~100,000	> 1,000,000
**Computational power required**	Low	Low	Medium/high (depending on the bioinformatic strategy)	High/very high (depending on the sequencing depth)
**Bioinformatics expertise**	Beginner/intermediate	Beginner/intermediate	Intermediate/advanced	Advanced/expert
**Taxonomic resolution**	Genus level (rarely species level for few microorganisms)	Species level	Species level	Species level (sometimes strains level with deep sequencing)
**Taxonomic coverage**	Bacteria and archaea	Bacteria and archaea	Bacteria, archaea, protists, and fungi (also viruses depending on the DNA extraction method)	Bacteria, archaea, protists, and fungi (also viruses depending on the DNA extraction method)
**Functional profiling**	No	No	No (but a little information can be retrieved)	Yes
**Genome reconstruction**	No	No	No (only genome portions of dominant microorganisms)	Yes
**Databases**	Ribosomal genes	Ribosomal genes	Marker genes or reconstructed genomes	Marker genes or reconstructed genomes
**Host DNA contamination (if any)**	No	No	Yes	Yes
**Amplification of contaminants (if any)**	Yes	Yes	No	No
**DNA alterations**	Yes (PCR artifacts such as chimeras and heteroduplex molecules)	Yes (PCR artifacts such as chimeras and heteroduplex molecules)	No	No
**Comparable data (with other projects)**	No (depending on the amplification region)	Yes	Yes	Yes
**Costs (based on reagent and equipment amortization)**	~50 USD	~80 USD	~80 USD	> 150 USD (price depend on sequencing depth)

An alternative methodology named shallow shotgun metagenomic sequencing has recently been developed to overcome the cost issue of deep shotgun metagenomic, focusing on sequencing a smaller amount of DNA from metagenomic samples^[12]^. Using the latter approach, the cost of the analysis is reduced and aligned with that of performing 16S rRNA microbial profiling, around 80 USD instead of hundreds of USD for deep shotgun sequencing. Notably, such shallow metagenomics is filling the gap between shotgun and 16S rRNA gene sequencing without losing the ability to retrieve a reliable taxonomic classification at the species level of each microorganism. In fact, it has been shown that the sequencing of 100,000 short reads, the depth usually used for shallow shotgun metagenomics, is the appropriate sequencing depth for classifying the microbial community at the species level with a solid statistical significance, instead of sequencing millions of reads in deep shotgun metagenomics^[13]^. Furthermore, shallow and shotgun metagenomic data can be shared within the scientific community to provide a feasible way to better compare public data. In this context, standardized metagenomic data can be used for *in silico* comparisons between multiple experiments, also called meta-analyses, to gain insights into the environmental dynamics among a huge number of samples that cannot be otherwise collected in a single study. Nonetheless, the implementation of pipelines and systems able to process shotgun data is essential to have a reliable overview of each microorganism inhabiting the sample.

Many tools have been developed focusing on classifying shotgun sequencing data using different alignment strategies. For example, basic local alignment and search tool (BLAST) is one of the most sensitive metagenomic alignment methods and, consequently, one of the most used software packages for DNA searches. On the other hand, BLAST is also computationally intense, resulting in time-consuming analyses. Thus, many tools aiming at profiling shotgun metagenomics data use different approaches to increase the speed of execution of analyzes, such as searching identical portions of DNA sequences (k-mers) or reducing the computational load with a marker-based classification. However, it has recently been proven that the use of a database composed of microbial marker genes does not provide a complete and accurate picture of the microbiome complexity^[14,15]^. This is correlated with the misclassification of a large portion of the sequenced DNA that cannot be classified if it does not explicitly belong to the unique genes of classified microorganisms. Thus, it is essential not to implicitly trust the profiling of such tools since very clean profiles showing few microorganisms can only summarize the actual complexity of the analyzed microbiome. In a sense, the currently ongoing competition in providing the fastest methodology to classify microbiomes can jeopardize the ability of the developed bioinformatics approaches to provide an accurate and reliable overview of the actual microbial biodiversity residing in a biological sample.

Another fundamental instrument for the classification of shotgun metagenomic data is correlated with the completeness of the database used to infer the microbial classification. If the database is filled with misclassified sequences, the output of the analysis will not be reliable. Furthermore, as mentioned above, if the database lacks many bacterial species, the resulting microbial profile will be an underestimation of the actual complexity of that microbiome. Moreover, the need for a continuous and proper update of databases used in metagenomic analyses should not be underestimated. Microbial taxonomy is in continuous evolution, and many changes can occur in a few months, providing an actual revolution in the classification of microorganisms. In this perspective, we would like to encourage the scientific community to investigate poorly characterized microbiomes through culturomics experiments to gain access to the genome sequence of novel microbial species not already discovered. In this context, it has been shown that unknown microorganisms, also referred to as microbial dark matter, can be easily found in unexplored environments, such as rural human populations, exotic animals, soils, and waters^[10,16]^. A fundamental step to uncover the complexity of microbiomes is to retrieve genomic sequences not already classified, for example, through the DNA sequencing of putative novel species identified using peptide mass fingerprints by matrix-assisted laser desorption ionization-time of flight mass spectrometry (MALDI-TOF MS)^[17]^. Limitations of MALDI-TOF MS technology are related to similarities between organisms and databases with a limited number of spectra, leading to poor discrimination between species. Besides, applying MALDI-TOF MS to discovering novel species is useful for enriching the database with additional spectra aiming to isolate these putative unknown microbial species. Thus, a constant update in the microbial taxonomy is crucial to provide reference genomes that will uncover the genuine complexity of microbial biodiversity for future metagenomic assays. This will also give an instrument to re-analyze the vast number of sequencing data collected in the last two decades.

To summarize, nowadays, it is essential to provide reliable metagenomic data that can be analyzed with comprehensive bioinformatics tools and, at the same time, that can be compared with other studies. The shotgun metagenomic methodology provides the complete repertoire of the microbial DNA within a sample, and, to reduce cost, a shallow approach can be applied without affecting the quality of the profiling results. It is also crucial to choose an adequate bioinformatics tool associated with a solid database that is progressively updated to minimize the number of misclassified microorganisms within the analysis. Additionally, shotgun metagenomic sequencing can be coupled with flow cytometry assays, qRT-PCR, or supported by synthetic chimeric DNA spikes added directly to environmental samples, allowing the estimation of the bacterial load of the analyzed biological sample. Thus, relative abundances assessed by bioinformatic pipelines can be finally converted into absolute values unveiling those microbiome dynamics that cannot be otherwise uncovered with standard profiling.

## DECLARATIONS

### Copyright

© The Author(s) 2022.
